# The influence of corporate social responsibility, ability, reputation, and transparency on hotel customer loyalty in the U.S.: a gender-based approach

**DOI:** 10.1186/s40064-016-3220-3

**Published:** 2016-09-13

**Authors:** Sung-Bum Kim, Dae-Young Kim

**Affiliations:** 1 College of Business Administration, Inha University, 421B Building 6, Incheon, South Korea; 2 Hospitality Management, University of Missouri, 115 Eckles Hall, Columbia, MO 65211 USA

**Keywords:** Corporate social responsibility, Corporate ability, Corporate reputation, Customer loyalty, CSR-related transparency, Gender difference

## Abstract

This research explored a conceptual framework incorporating interrelationships among corporate social responsibility (CSR), corporate ability (CA), corporate reputation (CR), and CSR-related transparency on customer loyalty within the hotel context. In this study, we also analyzed consumers’ propensity to support CSR initiatives through the socio-demographic indicator of gender. We used independent sample *t* test and multiple regression analysis to test hypotheses based on 487 responses from American participants. Four antecedents (i.e., CSR, CA, CR, and transparency) exhibited favorable effects on customer loyalty. Among these four factors, the positively perceived CSR initiatives had a greater impact on customer loyalty. In addition, according to our findings, female participants were more likely to have a positive perception of the four antecedents than males.

## Background

The hotel industry is one of the world’s fastest-growing business spheres (de Grosbois 2012), and it plays an indispensable role in the hospitality industry. According to a HospitalityNet report (2015), revenue from the hotel industry is expected to reach $550 billion U.S. dollars in 2016. The industry revenue was worth only $457 billion U.S. dollars in 2011; however, we experienced an increase in revenue of approximately $100 billion U.S. dollars over the past 5 years. Within the service industry, there is strong interest in determining the features that increase guest loyalty to particular hotels; it is commonly believed that loyal customers lead to higher profits. In today’s highly competitive market, researchers have investigated the factors that lead to customer loyalty (Pan et al. 2012), particularly within the hospitality and tourism industry (Yoo et al. 2011).

The role of corporate social responsibility (CSR) has been an important topic studied by both scholars and practitioners in recent decades. CSR is related to a company’s commitment to its societal obligations. Recently, researchers have begun to include CSR in loyalty behavior models. A socially responsible image can differentiate a brand and enhance customer loyalty. Corporate social responsibility helps to enhance sustainability of the hospitality industry and retain customers (Gao and Mattila 2014). Relevant studies have proposed that CSR directly generates customer loyalty (Martínez and del Bosque 2013). However, due to the fact that perceived CSR is a complex construct, some scholars are not convinced that customers will take it into account when they decide which hotel to choose.

Corporate Ability (CA) is also an important differentiator in this competitive business environment. CA is defined as “the company’s expertise in producing and delivering products and services” (Brown and Dacin 1997). Traditionally, quality in the hotel industry has been viewed as a primary feature for attaining a competitive lead and market distinction. The relationship between CA and customer loyalty has often been investigated in various service contexts (Zeithaml et al. 1996). Corporate Reputation (CR), one of the main factors related to any industry’s sustainability, has been linked to perceived CSR and corporate ability (Brown and Dacin 1997). Su et al. (2015) suggested that customer-perceived CSR events and CR are two intangible resources that might greatly benefit hotels. Recently, researches have shown the effectiveness of transparency in safeguarding the efficiency of companies’ CSR enterprises (Kang and Hustvedt 2014a, b). Transparency is defined as “visibility of and accessibility to data concerning industry practices” (Merriam-Webster 2010).

Building a loyal customer base is critical for a company’s growth (Kotler and Armstrong 2008). It is the foundation for sustainable competitive advantages (Dick and Basu 1994). The hospitality literature is full of investigations related to customer loyalty and its antecedents within the CSR literature (e.g., Martínez and del Bosque 2013). There are other factors in the literature that play an important role in attaining customer loyalty (Khan et al. 2015). Despite growing interest in CSR within the hotel industry, empirical support in this area remains limited. Current findings have shown that CSR and CA, CR and CSR-related transparency are fundamental concepts for explaining this important aspect. However, there have been no studies to investigate the integrated model that simultaneously measures the four variables’ (CSR, CA, CR, and CSR-related transparency) impact on customer loyalty in the hotel industry.

The relationship between CSR initiatives and consumer responses is moderated by factors that are specific to individuals. Researchers have emphasized the importance of gender in the market categorization process because it is a primary feature of customer decision-making (Ibrahim and Angelidis 1994). Yet, few researchers have examined the role of gender differences on the impact of CSR and related antecedents on consumer responses. Based on the disparities outlined above, the goal of this study was to explore the four antecedents (CSR, corporate ability, corporate responsibility and CSR-related transparency) of customer loyalty and determine how they influence customer loyalty simultaneously within the hotel context. In this study, we hope to explore how hotel customers’ perceptions of four factors differ and how this relates to customer loyalty by gender.

## Literature review

### Examples of CSR activities across industries

The purpose of CSR initiatives is to maximize the industry’s long-term favorable effect on society while minimizing the negative consequences (Petkus and Woodruff 1992). The examples of manufacturers and retailers pursuing CSR activities are numerous and include businesses as diverse as Target, Apple, Converse, Motorola, Emporio Armani, and The Gap. In the restaurant domain, McDonald’s sponsors its Ronald McDonald House Charities while Starbucks, Panera Bread, and Chipotle campaign for animal welfare. In the lodging industry, according to Kucukusta et al. (2013), hotels such as Wyndham Hotels and Resorts and Hyatt have made CSR an essential element of their strategic marketing plan. Customer skepticism about CSR involvement results from a lack of consumer awareness of such tangible community action outcomes more than from consumers’ doubt of company motives for pursuing such activities (Singh et al. 2009). Responding to consumers’ demands for more CSR activities and greater corporate transparency has been important in recent years (Saeidi et al. 2015). In line with agency theory, Piechocki (2004) argued that one essential feature of socially responsible entrepreneurship is transparent discourse with investors about CSR rules and events. Dubbink et al. (2008) have studied the role of transparency in safeguarding the effectiveness of many firms’ socially responsibility initiatives.

### Corporate social responsibility and corporate ability

Brown and Dacin (1997) refer to all the information about a company to which a consumer has access as a combination of both CSR and CA. Consumers make use of trade-offs between CSR product features and corporate ability (Pomering and Dolnicar 2009). To combat consumer apprehension about CSR involvement, companies can reinforce the effects of CSR if their products and services are believed to be of higher quality when these initiatives are adopted (He and Li 2011). To achieve this, hotel management must increase quality and preserve a maintainable improvement (Gray et al. 2000).

### Corporate reputation

To understand CR from a customer’s point of view, we drew on Walsh and Beatty’s (2007) definition of customer-based CR, or “the customer’s overall evaluation of a company based on his or her reactions to the company’s goods, services, communication activities, interactions with the company and/or its representatives or constituencies (such as employees, management, or other customers) and/or known corporate activities” (p. 129). Corporate reputations are perceived as the attributes that differentiate one firm from another (Barnett et al. 2006) or as reactions to the company’s services, communication activities, and interactions with the company and/or its representatives (Walsh and Beatty 2007). When confronted with a lack of personal experience regarding a product or the company’s civic action plan, individuals rely on the CR to judge its products as well as its intentions (Schnietz and Epstein 2005).

### Transparency

This phenomenon may reduce individuals’ information asymmetry, perceived risk, and skepticism by providing documents regarding where and under what conditions products are manufactured (Hustvedt and Bernard 2010). In some countries in the European Union corporate activities and CSR-related information are regularly issued to the public (van Wensen et al. 2011). However, elsewhere, including North America, reporting on CSR by companies is still voluntary with no repercussions if the companies refuse. Given the vulnerability of the economy, it is fundamental that establishments pursue customer loyalty and that accountability and transparency through public reporting are recognized (Belal et al. 2013).

### Customer loyalty

By generating and preserving customer faithfulness, a company develops long-term, mutually beneficial associations with its clients. A loyal customer base results in a maintainable competitive benefit (Mandhachitara and Poolthong 2011). By attracting loyal customers, executives can decrease advertising costs and diminish the impact of price sensitivity. Additionally, a high level of customer loyalty results in recommendations through positive word-of-mouth. Relevant studies have emphasized the significance of understanding the elements that contribute to customer loyalty. Due to the fact that more attention has been paid to the antecedents of customer loyalty, previous researchers have failed to provide consistent explanations regarding how they affect customer loyalty (Kumar et al. 2013). Therefore, the hospitality industry must focus on understanding customer needs in order to boost allegiance in an increasingly competitive market.

### The impact of CSR and CA on customer loyalty

Scholars have proposed that CSR directly generates more customer loyalty, without requiring the intervention of mediating variables (e.g., Martínez and del Bosque 2013). They claimed that corporate ability and CSR act as evaluative criteria for determining consumer purchase intentions. An assessment of a customer’s positive perception of CA leads to favorable customer behavioral outcomes (Zeithaml et al. 1996). Based on these findings, we posit the following hypotheses:

#### Hypothesis 1

Perceived CSR has a significantly positive influence on overall customer loyalty toward a hotel.

#### Hypothesis 2

Corporate ability has a significantly positive influence on overall customer loyalty toward a hotel.

### The impact of corporate reputation on customer loyalty

Corporate reputation has long been recognized as a significant source of competitive advantage (Ali et al. 2015). In this research, we defined and employ CR as an independent variable. Consequences of CR have been underexplored empirically (Walsh et al. 2009). Simply put, companies with good reputations are likely to attain more consumers. In addition, a favorable CR has been shown to positively affect behavioral outcomes of consumers (Gounaris and Stathakopoulos 2004). A company’s reputation can increase customer loyalty (Du et al. 2007). Therefore, we propose the following hypothesis:

#### Hypothesis 3

A favorable corporate reputation has a significantly positive influence on overall customer loyalty toward a hotel.

### The impact of CSR-related transparency on customer loyalty

Scholars have found that corporate transparency has significantly positive influences on consumers’ responses to a company (Bhaduri and Ha-Brookshire 2011). Customer loyalty is likely influenced by consumer’s perceptions of a company’s efforts to be transparent (Kang and Hustvedt 2014a, b). Based on the above discussions, we propose the following hypothesis:

#### Hypothesis 4

Corporate social responsibility-related transparency has a significantly positive influence on overall customer loyalty toward a hotel.

### Gender differences

Gender differences have been examined widely within the consumer behavior literature. Women and men play different roles and exhibit varying behaviors in society because they are socialized in different ways. Patino et al. (2014) endorsed the importance of gender as a key demographic factor for understanding consumers’ perceptions of CSR practices. Bossuyt and Van Kenhove (in press) found that women are more influenced by the ethical standing of companies than men. Rocha et al. (2005) argued that male and female perceptions of corporate ability differ significantly. Ma et al. (2014) found a marked moderating effect of gender in the relationship between customers’ perceived corporate ability and customer loyalty.

Researchers have recognized a positive association between gender and CR (Bear et al. 2010) in that males and females tend to utilize significantly different information processing strategies, which in turn influences judgment formation (e.g., Holbrook 1986). Hyllegard et al. (2012) argued that females are more likely than males to value comprehensive CSR data. Compared to men, women may be more likely to request publicly issued material related to CSR claims, which aid in their decision-making. Males are less influenced by information transparency (Bhaduri and Ha-Brookshire 2015). Meyers-Levy (1988) claimed that females allocate more importance than males to uncertainty, time and money constraints, and consequences of decisions and task factors. In light of these findings, we hypothesize that gender makes a difference in perceptions of CSR, CA, CR, and transparency within the hotel context:

#### Hypotheses 5

Compared to male respondents, female respondents perceive that CSR has a more positive influence on overall customer loyalty toward a hotel.

#### Hypotheses 6

Compared to male respondents, female respondents perceive that CA has a more positive influence on overall customer loyalty toward a hotel.

#### Hypotheses 7

Compared to male respondents, female respondents perceive that CR has a more positive influence on overall customer loyalty toward a hotel.

#### Hypotheses 8

Compared to male respondents, female respondents perceive that CSR-related transparency has a more positive influence on overall customer loyalty toward a hotel.

**Fig. 1:**
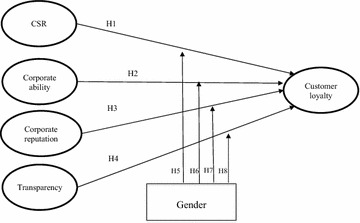
Research model

Based on these theoretical relationships among variables, we developed eight hypotheses. Our final conceptual model is shown in Fig. 1. This research demonstrates the relationships among the study constructs.

## Methods

### Measurement and survey questionnaire development

The questionnaire we selected for this research was designed based on established scales for all relevant constructs. We instructed all respondents to rate their perceptions of every item using a Likert-type scale to measure responses on a continuum of one for “strongly disagree” to five for “strongly agree.” Perceptions of CSR were measured via five items adapted from previous research (Brown and Dacin 1997). We adapted the three items for measuring corporate ability from Fombrun et al. (2000) and Brown and Dacin (1997). Customer perception of overall CR was measured using three items that were originally created by Weiss et al. (1999). We measured five transparency items via a scale designed to assess consumer perceptions of transparency, as outlined in previous studies (Hustvedt and Kang 2013). To measure customer loyalty, we employed a four-item scale from previous studies (Zeithaml et al. 1996). All items appear in the “Appendix”. Our questionnaire concluded with a series of demographic questions used to identify the respondent’s gender, ethnic background, age, level of education, and annual income.

### Data collection

We utilized a self-administered, online survey directed at customers in the United States called the Amazon Mechanical Turk because respondents are more demographically varied than other approaches, rendering this method a dependable means of enlisting participants for empirical inquiries (Rouse 2015). We established an incentive-based design in which the reward and incentive to contribute were directly linked to the tasks that the participants completed (Ding et al. 2005). As an incentive to participate in the survey, our respondents received a $.50 credit for their Mechanical Turk account. People who had stayed at a hotel within the last 6 months were encouraged to fill out the survey. Respondents were then given a link to our study’s webpage. We chose to utilize an online survey for cost savings, a rapid response time, greater control over the sample, and more effective data tabulation (Ilieva et al. 2002). The first part of the questionnaire included a description of hypothetical hotels and scenarios. We collected a total of 490 responses from participants.

### Data screening

Before conducting the data analysis, we examined the dataset for missing information. Because outliers may bias the mean and influence the normal distribution (Field and Hole 2003), participants who were unable to fill out the questionnaire completely were removed from the dataset prior to the data analysis. To locate outliers, we used descriptive statistics, box plots (Byrne 2001). Our study yielded a total of 487 complete and usable responses.

### Data analysis

First, we checked the reliability and validity of the measurement model. Then, we performed Confirmatory Factor Analysis (CFA), using AMOS. In order to study cause and effect, we employed a multiple regression model to evaluate the impact of the four elements on customer loyalty. Multiple regression analysis is the concurrent grouping of multiple factors to measure how and to what degree they affect a certain result (the causal relationships). We used an independent sample *t* test to calculate the mean differences in the four factors (CSR, CA, CR, and CSR-related transparency) between genders.

## Results

### Sample profile

The demographic profiles of the respondents are presented in Table 1. The cohort was 49.1 % male and 50.9 % female. Roughly half of the respondents (48.3 %) were between the ages of 25 and 34; the next largest age group consisted of individuals between the ages of 35 and 44 years (25.1 %). The respondents were primarily White/Caucasian (79.7 %), followed by Asian (7.0 %) and African American (6.4 %). Nearly 16 % of the respondents earned $30,000–39,999/year. Approximately 42.9 % of the respondents had some college education or an associates (2-year) degree.

**Table 1:** Profile of survey respondents (N = 487)

Demographic characteristics	Descriptive	Frequency	Percentage
Gender	Female	239	49.1
	Male	248	50.9
Age^a^	18–24	38	7.8
	25–34	235	48.3
	35–44	122	25.1
	45–54	52	10.7
	55–64	28	5.7
	Over 65	12	2.5
Education level	High school or less	61	12.5
	Some college or associate (2 year) degree	209	42.9
	Baccalaureate (4 year) degree	178	36.6
	Graduate studies/post-graduate studies	39	8.0
Race	White/Caucasian	388	79.7
	Asian	34	7.0
	African American	31	6.4
	Hispanic/Latino American	16	3.3
	American Indian/Native American	4	.8
	Pacific Islander	1	.2
	Other	13	2.7
Annual household income^b^ (2015)	$0–19,999	81	16.6
	$20,000–29,999	62	12.7
	$30,000–39,999	79	16.2
	$40,000–49,999	48	9.9
	$50,000–59,999	65	13.3
	$60,000–69,999	50	10.3
	$70,000–79,999	32	6.6
	$80,000–89,999	21	4.3
	$90,000–99,999	13	2.7
	Over $100,000	36	7.4

^a^Years old

^b^U.S. Dollars

### Confirmatory factor analysis of measurement scales

As shown in Table 2, findings from the CFA indicated that this model closely fit the data (*χ*^2^ = 1376.434, *df* = 303, *p* < .001), CFI = .90, IFI = .90, and RMSEA = .08). Factor loadings for the indicators for each construct were all significant (*p* < .01) and sufficiently high. To evaluate the internal consistency of the multi-item measures for each construct, we conducted a composite-reliability test for which all values ranged from .726 to .922, and were greater than the recommended threshold of .60 (Bagozzi and Yi 1988). Results suggested acceptable latent construct reliability. In terms of reliability, Cronbach’s alpha for all components was higher than .7 (Hair et al. 1998; CSR = .825, CA = .814, CR = .893, transparency = .853, customer loyalty = .780), indicating internal consistency within the measurement items. An acceptable level of convergent validity was evident in that all Average Variance Extracted (AVEs) were greater than .50 (Hair et al. 1998). We accessed the discriminant validity by comparing the AVE values with the square of the correlations between each pair of constructs, as shown in Tables 2 and 3. All investigated constructs met the discriminant validity requirements (Fornell and Larcker 1981).

**Table 2:** Confirmatory factor analysis: item measurement properties

Constructs	Items	Standardized factor loadings	Cronbach’s α	CR	AVE
CSR	CSR-1	.576	.825	.898	.643
	CSR-2	.663			
	CSR-3	.831			
	CSR-4	.815			
	CSR-5	.667			
CA	CA-1	.813	.814	.810	.756
	CA-2	.808			
	CA-3	.715			
Corporate reputation	CR-1	.849	.893	.897	.882
	CR-2	.878			
	CR-3	.846			
Transparency	TR_1	.709	.853	.726	.661
	TR_2	.729			
	TR_3	.753			
	TR_4	.729			
	TR_5	.780			
Customer loyalty	CL-1	.789	.865	.922	.798
	CL-2	.501			
	CL-3	.830			
	CL-4	.862			

Goodness-of-fit: *χ* ^2^ = 1376.434, *df* = 303, *p* < .001, RMSEA = .08, CFI = .900, NFI = .901, TLI = .902

*CSR* corporate social responsibility, *CA* corporate ability

**Table 3:** Construct intercorrelations

	1	2	3	4	5
1. CSR	1				
2. CA	.782	1			
3. CR	.696	.814	1		
4. TR	.800	.619	.587	1	
5. Customer loyalty	.737	.738	.640	.674	.726

*CSR* corporate social responsibility, *CA* corporate ability, *CR* corporate reputation, *TR* transparency

All coefficients were significant at *p* < .05

### Hypothesis testing

**Fig. 2:**
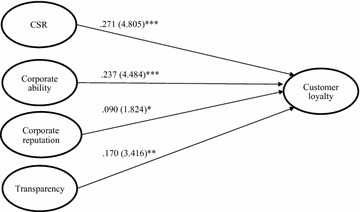
Multiple regression analysis results. *p < .05; **p < .01; ***p < .001 (one-tailed)

Overall CSR (*β*_CSR–customer loyalty_ = .271, t = 4.805, *p* < .001), CA (*β*_CA–customer loyalty_ = .237, t = 4.484, *p* < .001), CR (β_CR–customer loyalty_ = .271, t = 4.805, *p* < .05), and CSR related transparency (*β*_transparency–customer loyalty_ = .170, t = 3.416, *p* < .001). Therefore, hypotheses 1, 2, 3, and 4 were supported, as expected. The findings indicated that CSR, CA, CR, and CSR-related transparency (the independent variables) were generally associated with customer loyalty (the dependent variable). The results of the comparisons among the beta coefficients and the *t*-values revealed that CSR had a greater impact on customer loyalty toward a hotel. The regression results that we used to evaluate the hypotheses are presented in Table 4 and Fig. 2. Tolerance values ranged from .369 to .485. The variance inflation factor (VIF) was calculated, and the values for each independent variable in all regression models are smaller than 3.0, which indicated little multicollinearity (O’Brien 2007). Thus, the estimations were free of significant multicollinearity bias.

**Table 4:** Multiple regression analysis results

Regression model	Customer loyalty		
	Beta^b^	*t* values	VIF^a^
CSR	.271***	*4.805*	*2.711*
CA	.237***	4.484	2.379
CR	.090*	1.824	2.060
Transparency	.170***	3.416	2.122
R^2^ (Adjusted):			
Customer loyalty = .430			

All beta values are standardized. Italicized numbers indicate the values that have the strongest impact on the dimension

*CSR* corporate social responsibility; *CA* corporate ability, *CR* corporate reputation

* *p* < .05; ** *p* < .01; *** *p* < .001

^a^Variance inflation factor

^b^One-tailed test

**Fig. 3:**
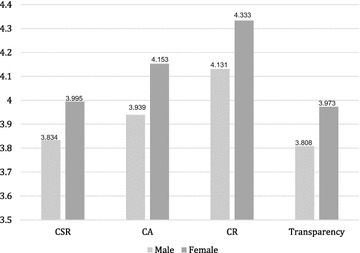
Gender difference in CSR, CA, CR, and transparency (male group and female group). *Note*: CSR: corporate social responsibility; CA: corporate ability; CR: corporate reputation

Then, we utilized an independent sample *t* test to link the mean scores of the four independent variables when comparing the responses of male and female respondents to further classify initial gender differences, as shown in Table 5 and Fig. 3. Levene’s test for homogeneity of variances was carried out. The results were of no significance (*p* > .05) for all the analyzed variables, so the assumption of variance equality was not rejected. The significant differences between the means of the two groups on perceived CSR, CA, CR, and transparency (*t*(485) = −3.114; *t*(485) = −4.175; *t*(485) = −4.074; *t*(485) = −2.921, *p* < .01). Overall, mean scores and standard deviations for all variables were higher among female respondents (CSR: *M*_female_ = 3.995 vs. *M*_male_ = 3.834; CA: *M*_female_ = 4.153 vs. *M*_male_ = 3.939; CR: *M*_female_ = 4.333 vs. *M*_male_ = 4.131; CSR related transparency: *M*_female_ = 3.973 vs. *M*_male_ = 3.808). Thus, hypotheses 5, 6, 7, and 8 were supported.

**Table 5:** Independent sample *t* test results between genders

Gender differences		Mean	SD	*t* value (two-tailed)	*P* value	Levene’s test	
						*F* value	Significance
CSR	Male	3.834	.560	−3114	.002**	.389	.533
	Female	3.995	.575				
CA	Male	3.939	.573	−4.175	.000***	.109	.742
	Female	4.153	.556				
CR	Male	4.131	.568	−4.074	.000***	1.046	.307
	Female	4.333	.522				
CSR related transparency	Male	3.808	.599	−2.921	.004**	.265	.607
	Female	3.973	.642				

*CSR* corporate social responsibility, *CA* corporate ability, *CR* corporate reputation

** *p* < .01; *** *p* < .001 (two-tailed)

### Conclusion and discussion

Attracting profitable customers and retaining them is always a key element of a successful business. Both those in the industry and academia are consistently seeking ways to increase customer loyalty. Although this is a popular topic, there has been no agreement on the factors that generate superior loyalty on the part of consumers (Mason et al. 2006) within the hotel context. Relevant studies have examined the antecedents and outcomes of customer loyalty (e.g., Xie and Chen 2014); however, there have been no studies examining the four elements’ link to customer loyalty within the hotel context. There have only been a few studies that have investigated gender difference among these four factors within the hotel context. Thus, the goals of this study were to explore the four antecedents of customer loyalty and determine how they influence customer loyalty simultaneously, and to investigate the variation of the perceptions of these factors between male and female customers within the hotel setting.

Our findings confirmed hypotheses 1 and 2. Our results highlighted the value of CSR initiatives in terms of customer allegiance and further support the findings of earlier studies (e.g., Galbreath 2010). It must be noted that He and Li (2011) had previously found a positive connection between CSR and customer loyalty. Hypothesis 2 is consistent with previous studies that have established the importance of quality management in the hospitality industry with the goal of increasing competitiveness (Wang et al. 2012). In addition to a company’s participation in CSR activities, service must be of high quality to elicit customer faithfulness (Mandhachitara and Poolthong 2011). We also confirmed that the contribution of CSR to a company’s attractiveness is much stronger than that of CA (Marin and Ruíz 2007).

In terms of hypothesis 3, although previous researchers have found that perceived CR is positively related to trust (e.g., Ganesan 1994) and the influence of CR on customer loyalty is fully mediated by customer satisfaction (Loureiro and Kastenholz 2011), their research confirmed the direct impact of CR on customer loyalty. With regard to hypothesis 4, our research showed that CSR-related transparency had a positive influence on overall customer loyalty toward the hotel, which is consistent with prior findings (Kang and Hustvedt 2014a). Organizations often keep a low profile with regard to their social responsibility activities, and they are reluctant to advertise their involvement for fear of customer dissatisfaction due to unfulfilled expectations (Schlegelmilch and Pollach 2005). With respect to transparency, Kelleher (2009) showed that companies need to consistently present thorough data to the public about their associations with social agencies. Therefore, it is vital for companies to reveal information openly and honestly in order to lessen charges of manipulation and “greenwashing” (Gray et al. 1995). It also indicates that providing transparent CSR information is an effective way to attract customer loyalty toward a hotel.

In terms of gender differences (hypotheses 5, 6, 7, and 8), whereas some researchers have found small or irrelevant changes (e.g., Trevino 1992) or even no significant association (Sikula and Costa 1994) in terms of perceived and CSR and customer loyalty. In fact a study by Bekkers (2010) indicated no gender gap within the CSR context. However, both male and female consumers reported that perceived CSR had a significant effect on customer loyalty. Our study confirmed along with some prior studies (O’Fallon and Butterfield 2005) that women are more likely than men to evaluate a company based on its CSR involvement.

Some scholars claimed that individuals’ perceptions of corporate ability do not vary with gender differences (Lee and Chen 2009). Mattila’s (2000) investigations of gender differences and consumer evaluations of service encounters yielded no significant differences between genders. Nonetheless, we confirmed that variations in the demographic profiles of customers can lead to variances in the perception of CA (Bekko 2000). According to Laroche et al. (2000), women also tend to rely more heavily on tangible and intangible cues in the service environment to make their evaluations. In addition, according to Ma et al., perceived corporate ability has a greater impact on females’ overall evaluations with a restaurant compared to males (2014). Lee et al. (2016) claimed that females have a stronger sense and awareness in terms of the overall service quality, while male’s abstract mind would be less conscious of the service quality and be more concentrated on the logical and consistent attributes of the industry.

Male and female participants respond differently in terms of the links between CR and customer loyalty. Based on our findings, we can infer that females are seen as comprehensive information processors, while male tends to be more selective and leave out subtle cues (Darley and Smith 1995). This indicates that women might be more likely to demand transparency with regard to CSR claims to aid in their decision-making process. This is consistent with previous findings that female customers place a higher value on the social and relational aspects of service (e.g., Iacobucci and Ostrom 1993).

## Theoretical implications

Our findings have several notable theoretical and practical implications. From a theoretical point of view, this research contributes to the hospitality industry’s goal of increasing CSR activities. Although CSR is a hot topic in most areas, such investigations in the hospitality and tourism sector lag behind those of other fields. Although a few investigators have examined the CSR practices of the hotel industry, their studies have been largely focused on environmental effects and performance (Benavides-Velasco et al. 2014). Liu et al. (2014) have proposed that our current understanding of the influence of CSR on customer response is still inadequate. While existing research in the hospitality industry has broadly explored loyalty issues, there have been few studies that focus on specifically on the concept of CSR’s relation to hotel customer loyalty.

Previous studies have revealed that the direct links of our independent variables (CSR, CA, CR, transparency) to customer loyalty have been inconclusive (e.g., Viada-Stenger et al. 2010). The studies directly examining the relationships between CSR, CA, CR, transparency and customer loyalty are still lacking within the context of the hotel industry. Thus, in this study, we have addressed research gaps in essential hospitality marketing areas.

Recently, researchers (Su et al. 2016) have pointed out that the impact of CR has been underexplored empirically. For example, Su et al. (2015) believed that only cursory attention has been paid to the standing of CR and CSR within the context of the hotel industry. Examining the association between CSR and CR is still a fairly new concept, so few scholars have studied it (Golob et al. 2013). Other researchers have argued that CR has an indirect effect on customer loyalty (e.g., Walsh and Wiedmann 2004). However, we found a direct relationship between CR and customer loyalty. This research will contribute to the hospitality literature because we found that respondents perceive that CR has a positive influence on overall customer loyalty toward a particular hotel.

While CSR initiatives have been shown to produce positive attitudes about a company, these attitudes may not translate into increased customer loyalty because consumers are unwilling to trade CSR for core attributes (Ailawadi et al. 2014). Most studies have found that CSR significantly influences CR; however, we argue that CSR and CR are independent variables. In other words, companies focus on CSR and CR simultaneously. This research will also add to the hotel CSR literature as a theoretical framework for assimilating these vital constructs. In particular, transparency has not been applied and tested in the hotel context.

Finally, while hospitality researchers have described gender similarities and differences with regard to consumption behaviors and perceptions, we found a difference between how men and women perceive these four independent variables (CSR, CA, CR, transparency). There is ongoing research on the presumed differences between men and women (De Wit and Bekkers, in press) with regard to purchase behaviors. Our study will contribute to this type of research by focusing specifically on the hotel context.

## Practical implications

From a practical perspective, while CSR efforts may simply be window dressing and propaganda, they do have a significant impact on customers—much like a marketing or advertising campaign. There has been discussion about whether hospitality companies should become involved in CSR activities (Saiia et al. 2003). According to Saiia et al. (2003), it has been suggested that hospitality managers should implement greater CSR involvement. Although this is now a worldwide trend in the hotel industry, hoteliers are largely unacquainted with this idea (Luck and Bowcott 2009). In terms of corporate ability, well-trained staff with good manners and a high level of expertise play a significant role in enhancing customer loyalty toward a service brand/company.

Companies must pay attention to the reputation, and they are well advised to carefully control the elements that contribute to this reputation (e.g., advertising) (Loureiro and Kastenholz 2011). By creating and developing a favorable CR, companies can benefit from higher levels of differentiation, appeal to investors, and obtain higher levels of customer loyalty. Given that our findings indicated that when consumers recognize companies as reputable, they indicate a higher level of loyalty, companies need to monitor CR on a regular basis to make appropriate adjustments.

The association between consumers and companies is influenced not only by the transparent actions of the company but also by consumers’ personal approximations of how the company reacts in circumstances in which its activities cannot be transparent (Kitchin 2003). Therefore, it is generally predicted by CSR advocates that socially responsible business entities should implement meaningful social and environmental initiatives and follow the values of transparency via public reporting of these initiatives and their efficiency (Sutantoputra 2009). Consumers who care about such transparency had more positive responses about socially responsible product claims, compared to non-transparent ads (Yan et al. 2012). To retain customer loyalty, hotel companies must share their socially responsible practices through public marketing messages. For instance, guides for sharing CSR efforts begin with the proposal that business executives “be credible, transparent, honest” about their companies’ actions (Middlemiss 2003).

From a demographic standpoint, our findings will help hospitality marketers to effectively target certain segments of the population and provide clearer insights into formulating more effective marketing strategies. Understanding how to successfully target individuals—irrespective of whether that approach is based on demographic characteristics—with appropriate message content is paramount. Thus, to attract/retain more customers, hotel marketers should provide a learning opportunity to this group of customers by delivering various information/knowledge about their programs/activities/services.

A loyalty program that benefits a charity can be a notable differentiator for an industry and produce considerable public relations benefits and opportunities for favorable publicity. These can be critical assets in today’s environment in which CSR activities are gaining more attention from customers. Since the business environment has become increasingly competitive, maintaining loyal customer relationships remains an essential element of business strategies. Thus, a loyalty program that can help a company distinguish itself, increase customer spending, and transmit a positive CSR message is essential to success (Eason et al. 2015). Such programs can help a company differentiate itself from others beyond the price of its products by providing customers with the opportunity to donate earned rewards to various charitable organizations. Not only will corporate social responsibility loyalty programs advance a company’s reputation and distinguish it from competitors, they will be enjoyed by customers. Finally, because consumers are one of the most significant stakeholder groups and are sensitive to a company’s CSR activities (Bhattacharya and Sen 2003), an understanding of stakeholder advantage is critical (Bhattacharya et al. 2009) to better comprehension of the outcomes of CSR activities. Because this research investigated consumer responses, it will help hospitality managers understand potential or existing customers.

In conclusion, corporate social responsibility enhances customer loyalty because consumers typically have positive responses toward companies that are socially accountable (Pomering and Dolnicar 2006); however, other scholars have found a distinct lack of consumer interested in CSR involvement (Vaaland et al. 2008). Despite its positive impacts, CSR activities may also inspire negative reactions. Yoon et al. (2006) discussed a backfire effect that results in an undesirable image and customers rejecting the company. Even if a company is associated with socially responsible agencies, it does not necessarily mean that a company is viewed as socially responsible (Mohr et al. 2001). We contend that CA, CR, CSR-related transparency, and CSR may be concurrently applied by hotel managers to develop an effective and valued company plan that provides sustainable competitive benefits.

## Limitations and future research

Our research has certain limitations which future researchers could work to overcome. For example, they could employ our model with a larger random sample or in other contexts. In addition, the representativeness of the samples collected from Amazon Mechanical Turk is debatable (Paolacci and Chandler 2014; Paolacci et al. 2010). Follow-up studies could transfer our model to various other service industries or other sectors of the hospitality industry. Future researchers might wish to replicate our research in other cultural contexts. The data for our research were collected using an online survey; however, future investigators should include a field survey to guarantee the practical applications of the data. For our study, we relied solely on self-reports; however, future researchers could employ a qualitative methodology to authenticate the model for a wider variety of participants and further test the model in these contexts. Longitudinal designs could be employed in the future to examine relationships over an extended timeframe, while our participants were surveyed only at a single point in time. Follow-up studies should include moderating and mediating effects within this context. It would be interesting to measure the connection between other socio-demographic characteristics and participants’ perceptions of CSR. Scholars could employ additional antecedents and consequences (e.g., actual behavior) to further develop the CSR literature and provide companies with competitive strategies. Because high-correlations among some of the independent constructs have shown (>.7), the estimations were not free of significant multicollinearity bias completely.

## Conclusion

This research contends that corporate ability and reputation, perceived corporate social responsibility, and CSR-related transparency should be concurrently applied by hotel executives to develop an effective and valued company plan that provides sustainable competitive benefits.

## Appendix

Scale items for construct measures

Corporate social responsibility

- This hotel seems to make an effort to create new jobs.
- This hotel would reduce its profits to ensure a clean environment.
- This hotel seems to be environmentally responsible.
- This hotel looks like a good company to work for.
- This hotel seems to treat its people well.

Corporate ability

- This hotel offers high quality products and services.
- This hotel is a strong, reliable company.
- This hotel develops innovative services.

Corporate reputation

- This hotel is highly-regarded.
- This hotel is very successful.
- This hotel is well-established.

Transparency

- Information about hotel’s CSR activities is easily accessible.
- It is easy to obtain sufficient information about hotel’s CSR activities.
- This hotel would be honest and sincere in addressing the environmental and societal issues.
- I can rely on this hotel to solve the problem of the environmental and societal issues.
- This hotel would make any effort to improve the environmental and societal issues.

Customer loyalty

- I usually use this hotel company as my first choice compared to other hotel brands.
- It would be costly in terms of money, time and effort to end the relationship with this hotel.
- I shall continue considering this one as my main hotel brand in the next few years.
- I would recommend this hotel if somebody asked my advice.
